# Effects of Watching Cartoons During an Echocardiography on Infants and Preschool Children. A Prospective Randomized Study

**DOI:** 10.3389/fped.2019.00184

**Published:** 2019-05-24

**Authors:** Francisco Sánchez Ferrer, M. Dolores Grima Murcia, Adriana Lopez-Pineda, Mercedes Juste Ruiz, Domingo Orozco Beltran, Concepcion Carratala-Munuera, Eduardo Fernández Jover

**Affiliations:** ^1^Pediatrics Department, University Hospital of San Juan de Alicante, Alicante, Spain; ^2^Institute of Bioengineering and CIBER-BBN. Miguel Hernandez University, Alicante, Spain; ^3^Catedra de Medicina de Familia, Clinical Medicine Department, Miguel Hernandez University, Alicante, Spain

**Keywords:** echocardiography, pediatrics, television, blood pressure, infants

## Abstract

Echocardiography is currently the main diagnostic technique in pediatric cardiology, but sometimes it is difficult to use in very young children, as a complete and accurate study depends on the patient's and family's cooperation. Children's behavior is one of the main problems for this procedure, and interventions like sedative medication have been used to facilitate its performance. The aim of this study was to analyze the effects of TV entertainment in infants and preschool children during echocardiography. We designed an experimental study in children with a heart murmur. An examination room was prepared with a TV on the ceiling, giving the children an unobstructed view during the echocardiography procedure. Fifty-eight patients were randomized into two groups: TV intervention vs. usual care (consisting of other distraction measures). The primary outcome was echocardiography time, but we also assessed blood pressure, quality of technique, child behavior, and parents' stress level. The TV group showed a statistically significant reduction in duration of the echocardiography and systolic and diastolic blood pressure, as well as better quality of technique and child behavior. Consequently, we recommend the use of a TV as a simple and useful distraction method for improving echocardiography in young children.

## Introduction

Echocardiography is a noninvasive procedure and is the first option for studying children with suspected congenital or acquired cardiac disease (1, 2). However, a complete and accurate study depends on patients' and their parents' cooperation.

The behavior of children under 3.5 years old sometimes makes it impossible to perform an echocardiography, prompting the application of interventions such as sedative medication (3). Even though chloral hydrate is one of the safest and therefore most frequently used procedures, it is not without risks. In order to quickly detect and treat adverse effects, the sedated patient must be continuously observed and monitored for all hemodynamic parameters, including oxygen saturation, heart rate, respiratory rate, and blood pressure (4). Adverse events affect anywhere from 0 to 20.1% of these children (5); in one study in 1,095 patients, observed events included apnea (0.3%), airway obstruction (1.4%), desaturation (5.9%), hypercarbia (5.9%), hypotension with poor perfusion (0.4%), vomiting (0.4%), and prolonged sedation (3.3%) (3). In a few cases (0.5%), major interventions were necessary to treat severe respiratory depression and/or hypotension with compromised perfusion. Moreover, the sedation medications may modify the patient's baseline physiological state by causing alterations in parameters such as heart rate and blood pressure or hemodynamic measurements (taken by echocardiography) (6, 7). Given the clear drawbacks of sedation, it is necessary to explore alternative interventions to facilitate echocardiography in pediatric patients. These could be more cost-effective and safer, reducing anxiety for the children and their families as well as for health professionals (8).

Some simple, readily available and cost-effective ways to reduce neonatal discomfort include giving the child a pacifier (dummy) or feeding them (9). In infants, other methods have been employed for minimally invasive procedures, such as watching cartoons (10), looking through kaleidoscopes (11), blowing bubbles (12), listening to music (13, 14), and wearing virtual reality glasses (15).

In children older than 3.5 years, previous authors have demonstrated that video goggles and earphones significantly decrease costs, the need for sedation, and scanning times in magnetic resonance imaging (MRI) and computerized tomography (CT) procedures, but they had no impact on the need for sedation in children younger than that (16). Lim et al. (10) used a children's song screened on a cell phone during an ultrasound examination (not echocardiography) in children aged 1–5 years old. The results showed that the cartoons were very helpful in 83% of patients and partially helpful in 10%; however, there was no control group. For their part, physicians have been shown to always consider and try to minimize parents' stress and anxiety during these procedures (17, 18).

We hypothesized that cartoons might be a useful distraction in the echocardiography procedure, helping to improve young children's behavior. Therefore, the present study aimed to analyze the effect of TV on children under 3.5 years old during an echocardiography.

## Materials and Methods

### Design

Randomized clinical trial (Trial Registration Identifier: NCT02498743).

### Setting and Subjects

This study included pediatric patients referred to a pediatric cardiology consultation for the first time, from 1 February to 31 May 2015, for evaluation of a heart murmur without any complexity. Pediatricians in the public consultation at our university hospital recruited participants. Inclusion criteria were: aged 6–43 months, an echocardiography requirement, and signed written informed consent from parents. Patients with any hearing or eye alterations were excluded. The Institutional Review Board approved this study, and all the children's parents provided informed consent.

After baseline assessment and before the echocardiography examination, each participant was randomly assigned to either the intervention or control group. A researcher used an online statistical computing program to generate permuted block randomized schema.

### Intervention and Control Groups

Patients were randomly allocated to two groups: the TV group watched cartoons with sound during the examination, and the control group received usual care, consisting of other distractions like a mobile phone with cartoons or videos, toys, music, songs sung by parents, caresses, or kisses. In both groups the patient had to be supine on the examination table during the echocardiography.

### Echocardiography Examination

**Figure 1:**
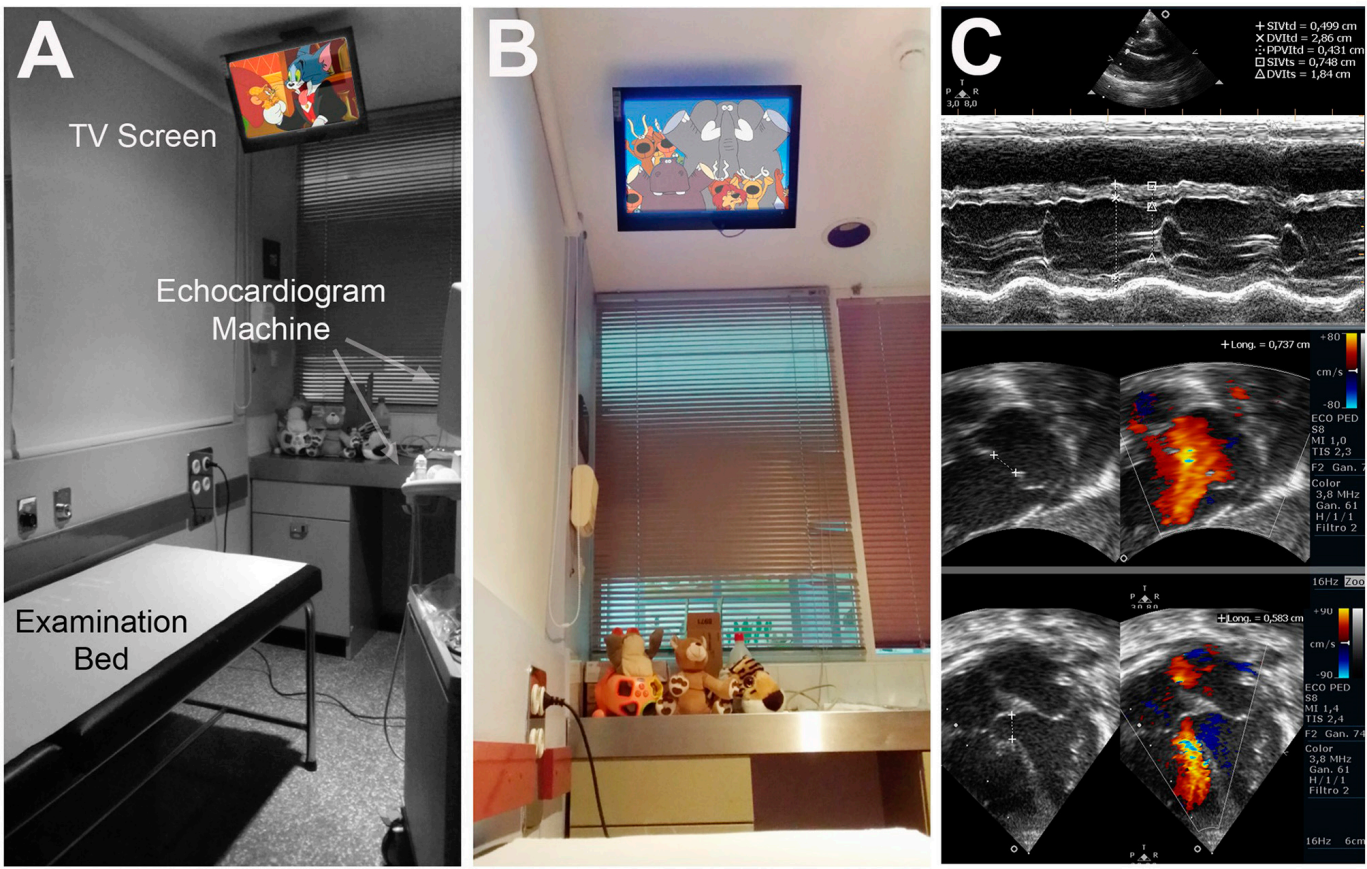
Children's examination room. **(A)** Detail of the examination bed and the TV screen on the ceiling. **(B)** Photograph from patient's point of view. **(C)** Sample echocardiographs while the patient was watching cartoons.

The patients' examination was performed in a room designed for this study. The cardiac ultrasound laboratory was equipped with a television on the ceiling, giving children an unobstructed view while they were laying comfortably in a supine position on the examination bed (see Figure 1). The TV was equipped with a conventional audio output and had a 19-inch monitor (DMTech).

The cartoon shown in this study was always the same (episode 125 of *Dora the Explorer*, “Baby Winky Goes Home!”). The episode was played on a continuous loop (automatically restarting again).

A single pediatric cardiologist conducted all consultations and performed the echocardiography with the help of the same assistant nurse in all cases and the child's parents, following current recommendations for performing transthoracic echography in children (19). The assistant nurse always wore the same work clothes, her attitude, and actions were similar in any case and she did not use any tools. The examination was performed using an ultrasound unit (Philips Envisor C HD) with S8 Mhz cardiac sector transducer. The blood pressure reading preceded the echocardiography and was taken with an automatic blood pressure monitor (Omron 10M-IT) with different children's sizes.

### Outcome Measures

The primary outcome was duration of the echocardiography and was measured by the assistant nurse. Date of birth and test date were collected from patients prior to randomization. After group allocation and before the examination, patients' blood pressure (mmHg) was measured by the assistant nurse with or without a TV, according to the trial arm.

Other outcome measures were recorded following the echocardiography: the quality of the echocardiography and the behavior of the child during the examination. The pediatrician performing the examination assessed the quality of the procedure (1 = poor, 2 = moderate, 3 = high); quality was considered high if all the echocardiography planes were performed completely and correctly, moderate if all the echocardiography planes were performed completely but not correctly, and low if the echocardiography was not completed and only partial planes were obtained. This assessment of the quality was considered impartial because this procedure is very methodical, since the echocardiography planes are established in a specific order. In addition, the difficulty of the echocardiography procedure was similar in all cases because no patient had indication for surgical correction of congenital heart disease. Children's behavior was assessed subjectively by both pediatrician and parents, who independently scored the child's behavior using a 10-point rating scale (1 = very bad, 10 = very good). The pediatrician answered only one question: “How did the child behave during the examination?” Parents answered six *ad hoc* questions about their stress level, their child's behavior, and their opinion of using cartoons as a distraction during the exploration.

### Statistical Analysis

Sample size calculation was based on the variable “duration of consultation” with an alpha level of 0.05 (95% confidence level), a beta level of 0.20 (80% statistical power), a variance of 4.84, and a margin of error of 1.6, assuming 15% attrition. Each group had to have at least 28 patients.

A descriptive analysis of the study sample was performed. We calculated the mean, minimum, maximum, and the standard error of the mean (SEM) for the study variables in each group. We applied the Student's *t*-test to compare the means and *p* < 0.05 was considered significant. Data were analyzed with the use of SPSS 18.0 for Windows.

## Results

Fifty-eight pediatric patients with a mean age of 26.03 months (range 6–43) underwent a consultation for an echocardiography procedure. None were excluded. Thirty patients (51.8%) were randomized to the intervention group (TV) and 28 patients (48.2%) to the usual care control (other distraction methods). Groups were comparable in terms of patient characteristics such as age and sex.

Table 1 shows the study variables in the overall sample. The scans lasted, on average, 9.05 min (range 6–16.6). The quality of the echocardiographs was moderate or poor in 31% of the scans. With regard to children's behavior, the doctor's and parents' assessments were similar: 64% of the patients received a score of 9 or 10, while 36% scored under 9. Regarding the question “Do you think that TV is useful?,” 85% of the parents answered affirmatively.

**Table 1:** Distribution of the study variables.

**Variables**	**Mean**	**Range**	**SEM**	**95% CI**
Age (months)	26.03	(6–43)	1.52	23.0–29.0
Duration of echocardiography (minutes)	9.05	(6–16.6)	0.27	8.5–9.6
Systolic blood pressure (mmHg)	94.6	(70–116)	1.41	91.8–97.4
Diastolic blood pressure (mmHg)	61.7	(43–82)	1.28	59.2–64.2
Children's behavior (0–10)
Physician assessment	8.00	(1–10)	2.64	2.8–13.2
Parent assessment	7.97	(1–10)	3.01	2.1–13.9
Quality of echocardiography (1–3)	2.55	(1–3)	0.73	1.1–4.0
Parents' stress level (0–10)	2.70	(1–10)	2.62	2.4–7.8

*CI, confidence interval; SEM, standard error of the mean*.

The time needed to complete the echocardiography was significantly lower in the TV group than in the control: 7.91 (7.51–8.33) min vs. 10.27 (9.55–11.13) min, respectively (*p* < 0.001). If 1,000 echocardiographs were performed every year, the total time needed, with or without TV, would be 7,910 vs. 10,270 min. Thus, using cartoons would entail 22.97% less time for the procedures, or 39.3 h/1,000 procedures.

Children's ages were similar in both groups. In relation to other variables (Table 2), the intervention group showed lower blood pressure values and better physician- and parent-reported behavior scores. Furthermore, the quality of the echocardiographs was significantly better in this group.

**Table 2:** Bivariate analysis.

**Variable**	**TV group mean (95% CI)**	**Usual care control mean (95% CI)**	***p*-value**
Age (months)	26.20 (22.00–30.35)	25.86 (21.74–29.88)	0.912
Systolic blood pressure (mmHg)	89.76 (86.87–92.93)	99.61 (96.08–103.27)	<0.001
Diastolic blood pressure (mmHg)	58.79 (56.00–61.93)	64.64 (60.58–64.58)	0.020
Children's behavior (0–10)			
Physician assessment	9.06 (8.48–9.53)	6.85 (5.66–8.02)	0.002
Parent assessment	8.77 (7.92–9.54)	7.11 (5.68-8.31)	0.038
Quality of echocardiography Scale (1–3)	2.83 (2.68–2.96)	2.25 (1.92–2.57)	0.003
Parents' stress level Scale (0–10)	2.24 (1.14–3.10)	3.18 (2.06–4.44)	0.191

*Results in intervention (TV) vs. control (no TV) groups*.

Parents' self-reported stress levels on a scale from 0 (no stress) to 10 (high stress) showed that 58% had no stress, but 20% had a stress level of 5 or more. Parents in the intervention group consistently registered lower stress levels, although the difference was not statistically significant (see Table 2).

## Discussion

Echocardiography is the most commonly used imaging procedure for cardiological evaluations in children (1, 2). However, its quality depends on patient compliance (2). Children may be irritable during the echocardiography [or other non-invasive procedures (20, 21)] for several reasons, including their lack of familiarity with the place, procedure and professionals; the darkness of the room; and their parents' attitudes. Even in non-invasive medical procedures without pain, like X-rays or ultrasounds (18), young children can still experience procedural distress that can interfere with diagnostic processes. This is especially true for echocardiography outcomes because this procedure requires not only an anatomical study but also a functional study. These reactions to medical situations have long been recognized (21), and many efforts have been made to identify ways to mitigate procedure-related distress in children.

The use of sedatives is one way to calm children down during examination procedures, but this option poses a risk for adverse events and even sedation failure. The cost of medication, plus other direct and indirect costs such as human resource expenditure and patient recovery time, must also be taken into account (3, 15). Moreover, sedation may cause anxiety in children and their parents. Nowadays, sedation is not recommended for an echocardiography in asymptomatic patients (22), but it is often needed in children with a high suspicion of cardiomyopathy to guarantee the quality of the echocardiography.

Alternative methods to improve child behavior during exploration have been reported (23), such as the use of pacifiers and sugar for neonates (24) and in children under 6 months of age (25). These patients were not included in the study because they have difficulty maintaining a fixed gaze. Another study in children older than 3.5 years, using an MRI-compatible movie entertainment system and interdisciplinary teams, showed a reduction in the frequency of patient sedation by 25% (15). A different study tried to calm infants and young children receiving an MRI, using a color light-show device projecting moving colored objects on the ceiling and floor, reporting that the need for sedation decreased by 29% (in those aged 1–2 years) and 19.9% (in those aged 2–3).

A study using children's songs on a cell phone showed positive results in 93% of patients, but it did not have a control group (10). Other studies have shown that pain tolerance is greater with the visualization of pleasant images (20, 26). In children aged 7–12 years old receiving venipuncture, using a TV was more effective as an analgesic method than no distraction or active distraction by mothers (27). A previous study also showed that the gradient values measured by the echocardiography in children watching cartoons corresponded well with catheterization values (6). Equally positive results have been achieved using music therapy as a support (8).

In our study, patients were randomized, and we measured different variables to compare the effect of television between the groups, avoiding other confounders. We measured the items that were considered to be of high interest in a cardiology clinic. Examination time decreased by an average of 2.4 min (*p* < 0.001), and the quality of the echocardiography was also significantly higher (*p* = 0.003). Thus, our method was helpful in young children (6–43 months).

Blood pressure was different between the groups. Measuring this parameter is not easy in children, as it is difficult for them to keep still. However, the intervention allows an approximation to the child's true blood pressure measurements. Systolic blood pressure was 10 mmHg lower, and diastolic blood pressure 6 mmHg lower, in the intervention group (with TV). Thus, using the television as a distraction method might also help to avoid the over-diagnosis of arterial hypertension in children (28).

Regarding the stress level during the echocardiography examination, parents self-reported their stress level on a scale of 0–10. The average score was low for both groups, but in the non-television group, it was slightly higher (not significant). This is important because the attitude of parents may influence their children.

In addition, in the intervention group some children did not want to leave the examination room once the echocardiography exploration had finished, suggesting that the patient's stress level was near zero. These positive experiences could have a beneficial impact on children's attitudes toward future explorations (29).

## Limitations

This study has some limitations. Children's behavior and parents' stress were assessed subjectively, and since we found no behavioral scales for non-invasive tests in children, we used a simple, *ad hoc* scale from 0 to 10. Another problem when performing diagnostic techniques is interobserver variability. In order to diminish this risk, a single pediatric cardiologist (in a hospital without a cardiac surgery pediatrician) performed this study. On the other hand, the control group received usual care, consisting of other distractions that were provided by parents. Although the behavioral attitudes of the parents can be different between them, this un-controlled factor affected both groups similarly.

## Conclusion

Showing cartoons on a TV to children undergoing an echocardiography procedure was effective in decreasing the examination time and the systolic and diastolic blood pressure, and it increased parents' and doctor's satisfaction with the procedure and the quality of echocardiography. This tool is also safe and avoids the added risk of anesthesia. The children and their parents were relaxed during the examination, and the children also showed signs that the echocardiography was an enjoyable experience. Thus, showing cartoons is a useful aid in children aged 6–43 months receiving an echocardiography.

## Ethics Statement

This study was carried out in accordance with the recommendations of Comite ético Hospital San Juan de Alicante with written informed consent from all subjects. All subjects gave written informed consent in accordance with the Declaration of Helsinki. The protocol was approved by the Comite ético San Juan de Alicante.

## Author Contributions

All authors contributed substantially to the realization of this article. FS is the principal investigator and conceived the study, acquired the data, interpreted and drafted the article. MDG designed the study room, analyzed the data, drafted and revised the final draft. AL-P, MJ, DO, CC-M, and EF conducted the study design, analyzed and interpreted the results, and drafted the article.

### Conflict of Interest Statement

The authors declare that the research was conducted in the absence of any commercial or financial relationships that could be construed as a potential conflict of interest.

## References

[1] Mertens L Friedberg MK. The gold standard for noninvasive imaging in congenital heart disease: echocardiography. Curr Opin Cardiol. (2009) 24:119–24. 10.1097/HCO.0b013e328323d86f 19225295

[2] Mertens L Seri I Marek J Arlettaz R Barker P McNamara P. Writing Group of the American Society of Echocardiography, European Association of Echocardiography, and Association for European Pediatric Cardiologists, Targeted Neonatal Echocardiography in the Neonatal Intensive Care Unit: Practice Guidelines and Recommendations for Training. J Am Soc Echocardiogr. (2011) 24:1057–78. 10.1016/j.echo.2011.07.014 21933743

[3] Heistein LC Ramaciotti C Scott WA Coursey M Sheeran PW Lemler MS. Chloral hydrate sedation for pediatric echocardiography: physiologic responses, adverse events, and risk factors. Pediatrics. (2006) 117:e434–41. 10.1542/peds.2005-1445 16481449

[4] Cote CJ Wilson S Coté CJ Wilson S Work Group on Sedation. Guidelines for monitoring and management of pediatric patients during and after sedation for diagnostic and therapeutic procedures: an update. Pediatrics. (2006) 118:2587–602. 10.1542/peds.2006-2780 17142550

[5] Napoli KL Ingall CG Martin GR. Safety and efficacy of chloral hydrate sedation in children undergoing echocardiography. J Pediatr. (1996) 129:287–91. 10.1016/S0022-3476(96)70256-1 8765629

[6] Stevenson JG French JW Tenckhoff L Maeda H Wright S Zamberlin K. Video viewing as an alternative to sedation for young subjects who have cardiac ultrasound examinations. J Am Soc Echocardiogr. (1990) 3:488–90. 10.1016/S0894-7317(14)80365-9 2278714

[7] Stevenson JG Kawabori I French JW. Doppler pressure gradient estimation in children. Accuracy, effect of activity and exercise, and the need for sedation during examination. Acta Paediatr Scand Suppl. (1986) 329:78–86. 10.1111/j.1651-2227.1986.tb10390.x 3473906

[8] DeLoach WD Procedural-support music therapy in the healthcare setting: a cost–effectiveness analysis. J Pediatr Nurs. (2005) 20:276–84. 10.1016/j.pedn.2005.02.016 16030507

[9] Stevens B Yamada J Lee GY Ohlsson A Sucrose for analgesia in newborn infants undergoing painful procedures In: Yamada J, editor. Cochrane Database of Systematic Reviews. Chichester: John Wiley & Sons, Ltd (2013). p. CD001069 10.1002/14651858.CD001069.pub4 23440783

[10] Lim SH Kim MJ Lee MJ. Use of animated cartoons with children's songs to increase compliance with ultrasonography in young children. Yonsei Med J. (2013) 54:1533–7. 10.3349/ymj.2013.54.6.1533 24142662 PMC3809887

[11] Vessey JA Carlson KL McGill J. Use of distraction with children during an acute pain experience. Nurs Res. (1994) 43:369–72. 10.1097/00006199-199411000-00009 7971302

[12] French GM Painter EC Coury DL. Blowing away shot pain: a technique for pain management during immunization. Pediatrics. (1994) 93:384–8. 8115196

[13] Hartling L Newton AS Liang Y Jou H Hewson K Klassen TP. Music to reduce pain and distress in the pediatric emergency department. JAMA Pediatr. (2013) 167:826–35. 10.1001/jamapediatrics.2013.200 23857075

[14] Arts SE Abu-Saad HH Champion GD Crawford MR Fisher RJ Juniper KH. Age-related response to lidocaine-prilocaine (EMLA) emulsion and effect of music distraction on the pain of intravenous cannulation. Pediatrics. (1994) 93:797–801. 8165081

[15] Harned RK II Strain JD. MRI-compatible audio/visual system: impact on pediatric sedation. Pediatr Radiol. (2001) 31:247–50. 10.1007/s002470100426 11321741

[16] Hoffman HG Doctor JN Patterson DR Carrougher GJ Furness TA. Virtual reality as an adjunctive pain control during burn wound care in adolescent patients. Pain. (2000) 85:305–9. 10.1016/S0304-3959(99)00275-4 10692634

[17] McMurtry MC Chambers CT McGrath PJ Asp E. When “don't worry” communicates fear: children's perceptions of parental reassurance and distraction during a painful medical procedure. Pain. (2010) 150:52–58. 10.1016/j.pain.2010.02.021 20227831

[18] Bradford R. Short communication: the importance of psychosocial factors in understanding child distress during routine X-ray procedures. J Child Psychol Psychiatry. (1990) 31:973–82. 10.1111/j.1469-7610.1990.tb00838.x 2246345

[19] Campbell RM Douglas PS Eidem BW Lai WW Lopez L Sachdeva R. ACC/AAP/AHA/ASE/HRS/SCAI/SCCT/SCMR/SOPE 2014 appropriate use criteria for initial transthoracic echocardiography in outpatient pediatric cardiology. J Am Coll Cardiol. (2014) 64:2039–60. 10.1016/j.jacc.2014.08.003 25277848

[20] Klosky JL Tyc VL Srivastava DK Tong X Kronenberg M Booker ZJ. Brief report: evaluation of an interactive intervention designed to reduce pediatric distress during radiation therapy procedures. J Pediatr Psychol. (2004) 29:621–6. 10.1093/jpepsy/jsh064 15491984

[21] Prugh DG Staub EM Sands HH Kirschbaum RM Lenihan EA. A study of the emotional reactions of children and families to hospitalization and illness. Am J Orthopsychiatry. (1953) 23:70–106. 10.1111/j.1939-0025.1953.tb00040.x 13016771

[22] Zilberman MV. How best to assure patient co-operation during a pediatric echocardiography examination? J Am Soc Echocardiogr. (2010) 23:43–5. 10.1016/j.echo.2009.11.007 20122494

[23] Birnie KA Chambers CT Spellman CM. Mechanisms of distraction in acute pain perception and modulation. Pain. (2017) 158:1012–3. 10.1097/j.pain.0000000000000913 28514252

[24] Slater R Cornelissen L Fabrizi L Patten D Yoxen J Worley A. Oral sucrose as an analgesic drug for procedural pain in newborn infants: a randomised controlled trial. Lancet. (2010) 376:1225–32. 10.1016/S0140-6736(10)61303-7 20817247 PMC2958259

[25] Edwards AD Arthurs OJ. Paediatric MRI under sedation: is it necessary? What is the evidence for the alternatives? Pediatr Radiol. (2011) 41:1353–64. 10.1007/s00247-011-2147-7 21678113

[26] de Wied M Verbaten MN. Affective pictures processing, attention, and pain tolerance. Pain. (2001) 90:163–72. 10.1016/S0304-3959(00)00400-0 11166983

[27] Bellieni CV Cordelli DM Raffaelli M Ricci B Morgese G Buonocore G. Analgesic effect of watching TV during venipuncture. Arch Dis Child. (2006) 91:1015–7. 10.1136/adc.2006.097246 16920758 PMC2082989

[28] Update on the 1987 Task Force Report on High Blood Pressure in Children and Adolescents: a working group report from the National High Blood Pressure Education Program National High Blood Pressure Education Program Working Group on Hypertension Control in Children and Adolescents. Pediatrics. (1996) 98:649–58. Available online at: https://pediatrics.aappublications.org/content/98/4/649.long?sso=1&sso_redirect_count=1&nfstatus=401&nftoken=00000000-0000-0000-0000-000000000000&nfstatusdescription=ERROR%3a+No+local+token 8885941

[29] von Baeyer C Marche T Rocha E Salmon K. Children's memory for pain: overview and implications for practice. J Pain. (2004) 5:241–9. 10.1016/j.jpain.2004.05.001 15219255

